# The Important Role of the Larynx in General Anæsthesia

**Published:** 1911-06-24

**Authors:** G. Beatson


### Hospital Clinics.

### THE IMPORTANT ROLE OF THE LARYNX IN GENERAL ANÆSTHESIA.

By SIR G. BEATSON, K.C.B., M.D.

In the production of general anæsthesia the risks from respiration are not the least of the dangers that beset the administration of any of the drugs employed for that purpose, it being an admitted fact that even while the heart is still beating the breathing may be arrested, either gradually or suddenly. In the former case the cessation is generally due to the toxic effects of the anæsthetic on the respiratory centre, whereas a sudden stoppage may arise from more than one cause. Thus, it may be due—(1) to falling back of the tongue; (2) to asphyxia from vomited matters or the entrance of blood; or (3) to that particular form of paralysis and adhesion of the vocal cords that renders inspiration impossible, and yet permits of a certain amount of expiration. This last state of matters is very misleading and deceptive, for during it the movements of respiration are going on normally or perhaps more vigorously than usual, and yet no air enters the chest, with the result eventually that the breathing suddenly stops in full expiration. There is, however, another form of sudden cessation of the respiration connected with the action of the anæsthetic on the highly-sensitive mucous membrane of the larynx that specially merits consideration, for it is a source of danger both in itself and through the heart, and, I think, furnishes an explanation of those unexpected deaths that take place with very little warning soon after the administration of the anæsthetic has begun. To make clear what I wish specially to draw attention to, I must refer briefly to the respiratory apparatus and to the nervous mechanism that works it.

### Some Practical Points.

Without going into details, the apparatus for breathing consists essentially of the larynx, trachea, bronchi, and two highly-specialised bodies the lungs, whose distinguishing feature is the elasticity of their tissue. Practically, they are two elastic bags, which can be enlarged so as to suck in air, and can then contract so as to drive it out, this variation in size being accomplished by that alternate expansion and contraction of the walls of the chest which take place in ordinary breathing. About the mechanism of these movements nothing need be said, but the following points should be remembered: (1) With the enlargement of the chest that accompanies inspiration a vacuum is formed within the lungs; (2) under ordinary circumstances this vacuum is filled chiefly by air, which rushes in to inflate the lungs, but also to some extent by blood, which enters the large venous trunks and the right side of the heart; (3) should there be any laryngeal or tracheal obstruction to the entrance of air, then the vacuum in the pulmonary alveoli tends to be filled by a transudation of fluid from the capillaries, producing a form of pulmonary œdema; (4) the amount of air that passes in and out of the respiratory apparatus during ordinary breathing (tidal air) seldom goes lower than the large bronchi; (5) the interchange of gases by which oxygen is introduced into the system and carbonic acid excreted takes place in the alveolar residual air of the lungs, and is carried out by the law of the diffusion of gases; (6) with ordinary respiration there are "associated respiratory movements" of (*a*) the nostrils and (*b*) the larynx, each of these structures dilating during inspiration and doing so by means of muscular action.

### The Respiratory Function.

Before considering the actual arrangement of the nervous mechanism of respiration, the three leading characteristics of the respiratory function should be borne in mind. In the first place, it partakes of the nature of an automatic act. Secondly, it is, in addition, partially controlled by will. Lastly, it is profoundly modified by reflexes. All these three features are familiar to us. We breathe more or less unconsciously, we can "hold our breath" or increase our respiration, and we all know well the uncontrollable reflex gasp that seizes us when our skin experiences the sudden contact of very cold water. It is this reflex element that plays such an important part in the normal respiratory process, and is very often responsible for disturbance of its rhythmic character.

### The Nervous Centre.

Coming now to the nervous mechanism itself, it has been definitely established that there is a portion of the medulla oblongata which must be intact to allow of the movements of respiration being carried on. Flourens, who first discovered it, called it "nœud vital," but, generally, it is spoken of as the "respiratory centre," it being, however, understood that it has two lateral halves, one for each half of the body, and is in reality a double "centre." Exception has been taken by some to the use of the word "centre," on the ground that respiratory impulses are not generated there, but this debatable question need not now be discussed. What is essential to bear in mind is that there is a certain limited portion of the medulla oblongata "at which the afferent nerves which call for respiration are brought into connection with all the various motor nerves which bring about the respiratory movements of the nostrils, larynx, chest, and diaphragm." (Hill.) In other words, it is the spot, or "centre," from which there arise automatically the rhythmical impulses necessary for the inspiratory acts, and to which there are also conveyed certain regular and irregular reflexes. That the latter should be present from time to time need create no surprise, for of all the vital centres the respiratory one is in closer touch with the whole of the body than any of the others. If any explanation of this be needed it is furnished by the fact that the respiratory tract has not only the power of warming, moistening, and purifying the air that enters the chest, but it has to furnish protection against the admission of any foreign bodies or irritating gases into the lungs. To enable it to perform this latter duty a portion of it has been specially adapted for the purpose. This is the part known as the larynx. It is placed at the upper end of the respiratory apparatus, just where it joins the alimentary canal, so that it is at a spot where it is common to both deglutition and respiration, and can guard the trachea against the entrance of particles of food and any pungent gases. As the vapour of several of the general anæsthetics in use, notably ether and chloroform, is of a strong and irritating nature, it is very important to understand the working of the larynx, otherwise the inhalation of the anæsthetic employed cannot be safely carried out.

### Inhibition.

The first point to bear in mind is that the ability of the larynx to afford protection to the trachea and lungs rests on the physiological fact that the glottis, through which the air finds its way into the lungs, can be partially closed, or even tightly shut, by certain of the laryngeal muscles. Possessed of this power, whenever any pungent vapour is inhaled the larynx either completely closes the glottis or tries to get rid of any fumes that may have got entrance to the trachea by the modified respiratory movements known as "coughing," or may even go a step further than this, and inhibit entirely all respiratory movements. The second point to remember is that all these different degrees of protection are carried into effect by reflex action, and that the nerves concerned are the pneumogastric or vagi. They are not the only afferent nerves which can influence the respiratory centre, but they are the ones most constantly active, and they come most into play through their superior laryngeal branches. It is these latter that supply the mucous membrane of the larynx and confer on it its great sensibility, and they can so materially affect the breathing that they are termed "the inhibitory nerves" of respiration, seeing that their stimulation can arrest the reflex action upon which the movements of the chest and diaphragm depend.

### The Role of the Larynx.

From what has just been said it will, I think, be quite evident that the larynx must play a very important rôle in the administration of any anæsthetic possessed of an irritating vapour, for its use in too concentrated a form may induce closure of the glottis, or coughing, or even complete arrest of respiration—any one of these conditions seriously interfering with the success of the anæsthesia. Hence it may be taken as an invariable rule that at the commencement of the administration of any pungent anæsthetic the whole attention should be concentrated on the management of the larynx, and that the aim should be to anæsthetise it first, so as to allow of there being given later these larger doses of the vapour by which alone general anæsthesia can be attained. This preliminary anæsthetising of the larynx can only be accomplished (1) by the local action of the anæsthetic and (2) by whatever vapour finds entrance to the blood from the lungs. If no vapour gets access to these organs owing to spasm of the larynx, or coughing, or arrest of breathing, then no progress is made with that dulling of the laryngeal mucous membrane which is so essential for obtaining a quick and safe general anæsthesia.

### Some Guiding Rules.

Attention to the following rules will, I know from experience, materially assist in overcoming the laryngeal sensibility:—

1. Remember that the object aimed at by giving the anæsthetic is to send the patient to sleep. This should be taken as the guiding principle in everything that is done in the first steps of the administration, and for it there is the highest authority, as in the first case of surgical general anæsthesia on record the Biblical narrative tells us that a deep sleep fell upon Adam. The French, too, recognise the same fact, as the word in use by them for anæsthetising anyone is "endormir": to send to sleep. Taking this, then, as a guiding principle or, rather, working basis, it follows that the position of the patient should be one of comfort, and should be the attitude preferred by the individual when he or she lies down to rest every night. This, of course, varies. Some like to sleep with the head raised, and others with it low, but preference should be given to the one in daily use, unless it is one very much out of the common, for, of course, all unnatural positions of the head are barred in administering an anæsthetic. This point settled, attend next to what is done by most individuals before retiring to rest—that is, the removal of any artificial teeth from the mouth. Lastly, as far as possible get the patient freed from all grounds of apprehension or alarm, for a person unduly anxious is not favourably placed for going to sleep. Perhaps the most common cause of dread is the fear of being suffocated, a feeling best dispelled by the assurance that plenty of air will be given and that the anæsthetic will be very sparingly used at first. With the mind so relieved, the patient to be anæsthetised will feel more at ease, and be better placed for facing what can only be described as a trying ordeal.

2. In your administration carry the patient with you. This may be done by a brief word of explanation as to what the administrator wants the patient to do, and by pointing out what great assistance may be given by quiet and steady breathing or by slow and regular counting aloud. Obtaining in this way the co-operation of the patient is exceedingly valuable, and yet means are seldom taken to explain to the individual to be anæsthetised any of the steps of the procedure, so that there is eliminated a factor of very great importance.

3. The administrator should use a drop bottle for the anæsthetic, and should sprinkle only a few drops on whatever apparatus is used, being very careful to see that there is a proper admixture of atmospheric air with the vapour and that it is so diluted that breathing or counting can be comfortably carried on. If there is the slightest interference with either, the apparatus used should be withdrawn and the patient allowed an inhalation of pure air. Comfortable and free inspiration is essential for safe anæsthesia. The point to bear in mind is that it is not the amount of anæsthetic poured on the apparatus that induces narcosis, but what gains admission to the lungs, and that when there the vapour makes its way to the air-cells by diffusion, a not very rapid process, and one that cannot be hurried. It has been, perhaps, too much lost sight of that Snow showed conclusively that eighteen minims of choloroform slow-progressively administered will produce in an adult loss of sensation, consciousness, and motion, all that is required for any ordinary surgical operation.

4. Avoid, too, in this early stage of anæsthesia, all meddlesome manipulations about the mouth and jaw. There should be no swabbing out of the mouth, and no unnecessary manipulations of the jaw. There should be an entire absence of meddlesome anæsthesia, which is not in keeping with sending anyone to sleep. As long, too, as counting is continued there is no need to test the lid reflex.

With careful attention to the above points the initial difficulties connected with the larynx will be obviated, and the way opened to a successful anæsthesia. On the other hand, neglect of them may delay the anæsthesia, or may introduce into it complications and even serious disaster. Thus it is well known that the proximity of the respiratory and vomiting centres in the medulla may lead to the occurrence of the latter act, for irritation of the respiratory centre often induces nausea or even actual vomiting, a very troublesome complication during the induction of anæsthesia. Through the superior laryngeal branch of the vagus such irritation could be easily conveyed. What, however, is of greater consequence is that the sudden closure of the glottis that may be induced reflexly through the laryngeal irritation of a strong dose of a pungent anæsthetic may be fraught with great peril to the patient in another way—namely, by indirect action on the heart. This risk is associated with the fact pointed out by Huxley that if a strong expiratory effort is made when the lungs are distended, and the entrance to the air passages is closed, the heart's action may be stopped altogether. So in the same way, if the lungs being partially emptied and the entrance of air prevented, a strong inspiratory effort is made, a similar result may take place. In the first case, as Huxley says, the reason of the stoppage is not very clear, but under the second condition the excessive distension of the heart, in consequence of the flow of blood into it, may be the cause of the heart's arrest. In either case, it is quite apparent that a very anxious situation is created, and it is quite possible that it may furnish the explanation of those sudden and mysterious deaths that from time to time happen in the very early stage of the administration of an anæsthetic. It is on these grounds that I hold that the slow and gradual anæsthetisation of the larynx is the matter that claims the whole attention in the beginning of the procedure, and that the above lines are the ones to work upon to ensure safe and quick results from a general anæsthetic.

